# Heterologous Biosynthesis of Crocin I in *Solanum lycopersicum* L.

**DOI:** 10.3390/ijms26209984

**Published:** 2025-10-14

**Authors:** Lei Xie, Jingjing Liao, Chongnan Wang, Xunli Jia, Yimei Zang, Changming Mo, Xiaojun Ma, Zuliang Luo

**Affiliations:** 1 State Key Laboratory for Quality Ensurance and Sustainable Use of Dao-Di Herbs, Institute of Medicinal Plant Development, Chinese Academy of Medical Sciences and Peking Union Medical College, Beijing 100193, China; leixie1996@163.com (L.X.); cnwang@implad.ac.cn (C.W.); xunlijia1998@163.com (X.J.); meiyee0810@sina.com (Y.Z.); 2 State Key Laboratory for Quality Ensurance and Sustainable Use of Dao-Di Herbs, Artemisinin Research Center, Institute of Chinese Materia Medica, Beijing 100700, China; jjliao@icmm.ac.cn; 3 Guangxi Crop Genetic Improvement and Biotechnology Laboratory, Guangxi Academy of Agricultural Science, Nanning 530007, China; mochming@126.com; 4 Yuelushan Laboratory, Changsha 410128, China

**Keywords:** crocin I, *S. lycopersicum*, synthetic biology, plant chassis

## Abstract

Crocins are high-value apocarotenoid pigments with broad applications in pharmaceuticals, foods, and personal-care products, and they exhibit diverse bioactivities, including antioxidant, antidepressant, and antidementia effects. In this study, we achieved the heterologous biosynthesis of crocins in *Solanum lycopersicum* L. by introducing the *GjCCD4a*, *GjALDH2C3*, *GjUGT74F8* and *GjUGT94E13* of *Gardenia jasminoides* J.Ellis to the binary expression vector via in-fusion technology and self-cleaving 2A peptides. Following *Agrobacterium*-mediated transformation, the engineered tomato plants predominantly produced main active ingredient crocin I, which accounted for 97–99% of the total crocins. The transgenic fruits displayed mixed red-and-golden colouration. These results highlight *S. lycopersicum* as a promising chassis for crocin I biosynthesis, helping to address supply constraints and enabling colour-trait breeding through synthetic biology.

## 1. Introduction

Crocins are water-soluble apocarotenoid pigments first discovered in saffron. The fruit of *Gardenia jasminoides* J.Ellis, a plant used both medicinally and as food [1], also accumulates crocins. These compounds have been reported to exhibit a range of health benefits, including antidepressant [2], anti-inflammatory [3], hepatoprotective [4], and neuroprotective effects [5], as well as therapeutic potential in Alzheimer’s disease [6]. However, due to limited resources, crocins currently suffer from high costs, which restricts their clinical research and application, as well as food development. Additionally, crocins are unstable and prone to degradation under factors such as high temperature, light exposure, and pH variations [7,8]. This instability further limits their applications, while the challenges in preservation further drive up costs.

**Figure 1:**
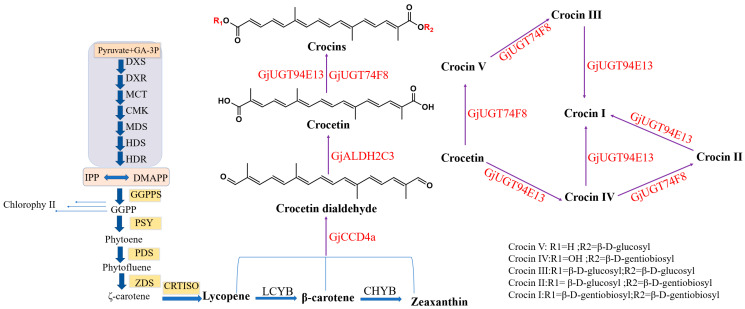
Biosynthetic pathway of crocins in the fruits of *G. jasminoides*. The enzyme expressed by the gene on the recombinant expression vector is marked in red. GjALDH2C2, aldehyde dehydrogenase; GjCCD4a, carotenoid cleavage dioxygenases; GjUGT94E13 and GjUGT74F8, UDP glucosyltransferases; MEP, methylerythritol 4-phosphate; IPP, isopentenyl diphosphate; DMAPP, dimethylallyl diphosphate; GGPP, geranylgeranyl pyrophosphate; GPPS, geranyl diphosphate synthase; FPPS, farnesyl pyrophosphate synthase; GGPPS, geranylgeranyl diphosphate synthase; PSY, phytoene synthase; PDS, phytoene desaturase; ZISO, ζ-carotene isomerase; ZDS, ζ-carotene desaturase; CRTISO, carotenoid isomerase; LCYE, lycopene ε-cyclase; LCYB, lycopene β-cyclase.

Carotenoids are produced in plastids and serve as photoprotective pigments in photosynthesis, while crocins are synthesized using carotenoids such as lycopene, β-carotene, and zeaxanthin as substrates. The main substrates of carotenoids come from the methylerythritol 4-phosphate (MEP) pathway, but the isopentenylpyrophosphate (IPP) and dimethylallyl pyrophosphate (DMAPP), produced by the mevalonic acid (MVA) pathway in the cytoplasm, can enter plastids [9]. DMAPP and IPP, mainly from the MEP pathway, react to generate geranylgeranyl pyrophosphate (GGPP). Phytoene synthase (PSY) is the rate-limiting enzyme that catalyzes the conversion of GGPP to phytoene. Then, lycopene is generated under the catalysis of phytoene desaturase (PDS), ξ-carotene isomerase (Z-ISO), ξ-carotene desaturase (ZDS), and carotenoid isomerase (CRTISO) [10]. This process further generates another two carotenoids: β-carotene and zeaxanthin (Figure 1). Carotenoid cleavage dioxygenase (CCD) of *G. jasminoides* can utilize lycopene, β-carotene, and zeaxanthin to produce crocetin dialdehyde and oxidized to form crocetin under the action of GjALDH2C3. GjUGT74F8 can use crocin IV as a substrate to generate crocin II, while GjUGT94E13 can glycosylate crocetin, crocin III, and crocin IV to produce crocin I [11]. Carotenoids are protective agents of photosynthesis and widely exist in various plant tissues [12]. The synthesis of crocins through plant chassis may be an important way to promote its application and solve the shortage of resource problems. Its primary components, crocin I and crocin II, are the detection markers specified in the *Chinese Pharmacopoeia* [13] and represent the main active constituents of saffron [14,15,16,17]. While crocin I is the most abundant crocin component found in saffron and *Gardeniae Fructus*, the separation and purification of crocin I has remained a subject of considerable scientific interest [18,19]. Perhaps by means of synthetic biology, we could simplify the purification process of crocin I.

The carotenoid cleavage dioxygenase GjCCD4a from *G. jasminoides* exhibits higher catalytic activity than the saffron ortholog CsCCD2L in *Nicotiana benthamiana* [20]. When G. jasminoides’ crocin biosynthetic genes were expressed in *Nicotiana benthamiana*, crocin I accounted for up to 84% of the total crocins, and crocin II reached up to 75% in the T1 transgenic *Nicotiana benthamiana* lines [11]. *Nicotiana benthamiana* harbours endogenous glycosyltransferases capable of catalyzing crocetin, but these enzymes preferentially facilitate the synthesis of crocin II. As a result, previous studies have typically reported higher levels of crocin II than crocin I in transgenic systems [21,22,23]. Tomato (*Solanum lycopersicum* L.) is an excellent candidate chassis for synthesizing crocin I. There is no conclusive evidence that endogenous glycosyltransferases catalyze the synthesis of crocin II, and it is one of the most widely cultivated and consumed vegetables globally. Furthermore, because crocins are chemically unstable and degrade upon processing, the fresh consumption of tomatoes can help preserve the bioactivity of crocins. In addition, tomato cultivation covers a wide area and generates a large amount of agricultural waste from nonedible parts. If constitutive promoters are used to drive the expression of crocins, the nonedible parts can be used for saffron extraction to solve the resource problem of crocins. Moreover, colour is a key determinant of *S. lycopersicum* fruit quality, and breeding for novel colours has always been a major focus of research; crocin accumulation could contribute desirable hues that enhance consumer appeal [24,25].

In this study, we constructed a vector containing four genes for the synthesis of crocins from *G. jasminoides*, including *GjCCD4a*, *GjALDH2C3*, and *GjUGT74F8* and *GjUGT94E13*. These genes were expressed in *S. lycopersicum* plants (*S. lycopersicum* cv. ‘Micro-Tom’) under the control of combinatorial promoters. The resulting transgenic *S. lycopersicumes* were capable of synthesizing crocin I, which constituted ≥97% of the total crocins. Additionally, crocins were detected in the stems and leaves of transgenic plants, offering a novel source of raw material for crocin extraction and contributing to the resolution of resource limitations.

## 2. Results

### 2.1. Multigene Vector Construction and Transient Expression Assays

**Figure 2:**
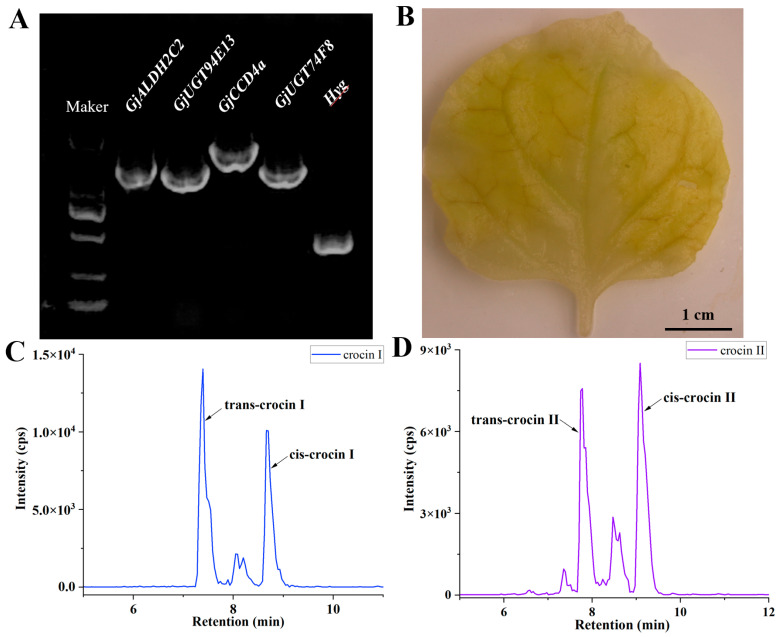
Transient transformation of *Nicotiana benthamiana* leaves with AU-CU vector. (**A**) PCR identification results of *GjALDH2C2*, *GjUGT94E13*, *GjCCD4a*, *GjUGT74F8*, and *Hyg* on the AU-CU multigene vector. The DNA marker is 2000 bp. (**B**) Phenotype of crocins accumulation in transiently transformed *Nicotiana benthamiana* leaves. (**C**,**D**) Extracted ion chromatograms of crocin I (**C**) and II (**D**) detected by HPLC-MS/MS in transiently expressed *Nicotiana benthamiana* leaves.

After three rounds of vector construction, the four genes involved in crocins biosynthesis were successfully assembled into the pCAMBIA1300 vector (Figure S1). PCR amplification of the four biosynthetic genes, along with the hygromycin resistance gene, confirmed the successful construction of the multigene vector (Figure 2A). To verify the functionality of the assembled vector, transient expression assays were conducted in both *Nicotiana benthamiana* and *S. lycopersicum*. Following the methods established in our preliminary studies, leaf samples from *Nicotiana benthamiana* and fruit samples from *S. lycopersicum* were collected 5 days post-infiltration for HPLC-ESI-MS/MS analysis. As shown in Figure 2C,D and Figure S2, the main active compounds crocin I and crocin II accumulated in both *Nicotiana benthamiana* leaves and *S. lycopersicum* fruits tissues transformed with the AU-CU multigene expression vector. Notably, after ethanol decolourization, *Nicotiana benthamiana* leaves exhibited a golden colouration attributed to crocin accumulation (Figure 2B).

### 2.2. Genetic Transformation and Molecular Identification

**Figure 3:**
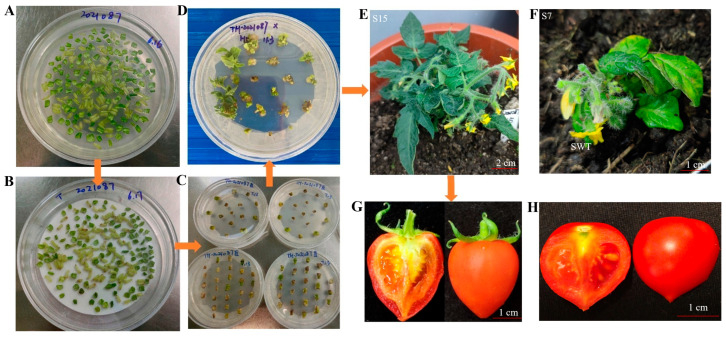
Process of Agrobacterium tumefaciens-mediated transformation of multigene Vector AU-CU into *S. lycopersicum*: (**A**) pre-culture; (**B**) co-culture; (**C**) differentiation and selection culture; (**D**) adventitious bud formation; (**E**,**F**) regenerated plantlet culture; (**G**) transgenic tomato fruit; (**H**) wild-type tomato fruit.

*S. lycopersicum* is one of the most widely consumed vegetables globally, and its predominant pigment, lycopene, serves as a substrate for GjCCD4a, enabling heterologous crocin biosynthesis while potentially modifying fruit colour. In this study, cotyledons and hypocotyls were used as explants for genetic transformation, with hypocotyls exhibiting the higher regeneration rate. The transformation process is illustrated in Figure 3. Notably, some hygromycin-resistant seedlings displayed an albino phenotype (Figure S3).

**Figure 4:**
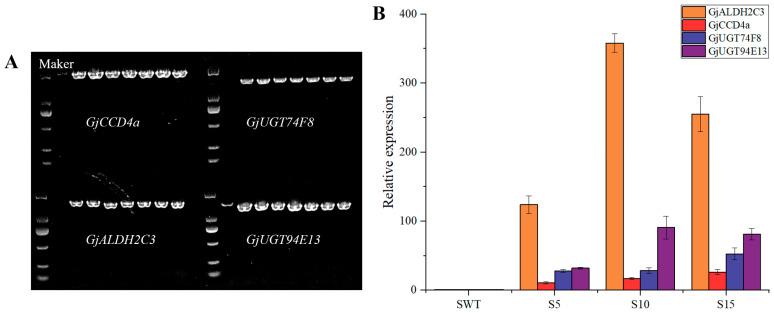
Molecular identification of transgenic *S. lycopersicum*. (**A**) PCR detection of crocins biosynthesis genes. The first swimming lane was a 2000 bp marker, and the second lane was the wild type, followed by transgenic *S. lycopersicum* lines S5, S7, S10, S13, S15, S24, and S25 from right to left. (**B**) Analysis of relative expression levels of crocins biosynthesis genes in transgenic *S. lycopersicum* lines S5, S10, and S15. *Leactin* was used as the internal reference gene, with the expression level of the wild type (SWT) set to 1. The data are presented as the mean values ± SDs; n = 3 represents three biologically independent samples.

A total of seven transgenic *S. lycopersicum* lines were confirmed by molecular identification and designated S5, S7, S10, S13, S15, S24, and S25 (Figure 4A). Among these, lines S7 and S13 exhibited golden-yellow leaves, while the other lines showed no obvious phenotypic changes. However, both S7 and S13 failed to survive post-transplantation due to poor initial growth and subsequent fungal leaf spot disease. Consequently, only PCR validation and qualitative crocin detection were performed on their leaves. Of the five surviving transgenic lines, only three non-albino plants (S5, S10, and S15) successfully produced fruit. As shown in Figure 3, although fruit colouration was predominantly masked by endogenous lycopene, the transgenic *S. lycopersicum* fruits exhibited a golden-red hue compared to the non-transgenic controls. The qRT-PCR analysis revealed that *GjALDH2C3* had the highest relative expression among the four transgenes, followed by its tandem gene *GjUGT94E13*, while *GjCCD4a* and *GjUGT74F8* showed lower expression levels. Notably, line S10 exhibited the highest overall transgene expression, whereas S5 had the lowest (Figure 4B).

### 2.3. HPLC-MS/MS Analysis

**Figure 5:**
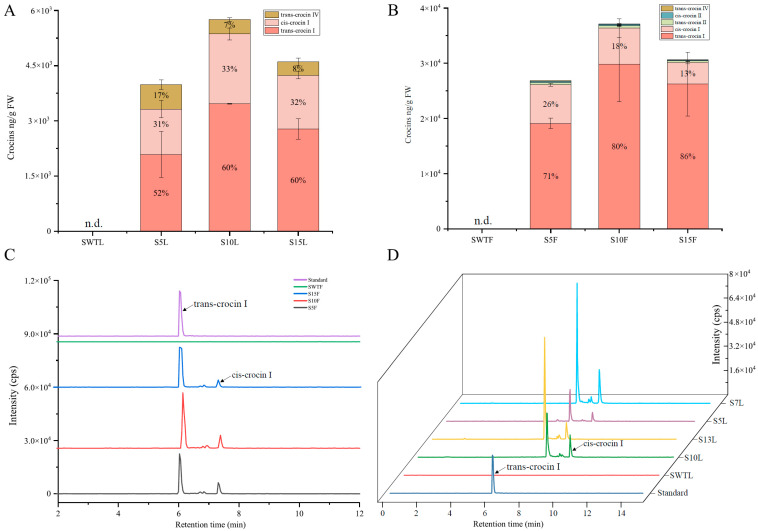
HPLC-MS/MS analysis of transgenic *S. lycopersicum*: (**A**) analysis of crocins accumulation in leaves of transgenic *S. lycopersicum* lines S5, S10, and S15; (**B**) analysis of crocin accumulation in fruits of transgenic *S. lycopersicum* plant lines S5, S10, and S15; (**C**) HPLC-MS/MS extracted ion chromatogram of crocin I in fruits of transgenic *S. lycopersicum* plant lines S5, S10, and S15; (**D**) HPLC/MS-MS extracted ion chromatogram of crocin I in leaves of transgenic *S. lycopersicum* plant lines S5, S10, S7, and S13. The data are presented as the mean values ± SDs; n.d.: not detected. n = 3 represents three biologically independent samples.

Among the seven obtained transgenic *S. lycopersicum* lines, only three lines—S5, S10, and S15—successfully set fruit; HPLC-MS/MS analysis was performed on both leaves and fruits of these lines. In the leaves, only crocin I and trans-crocin IV were detected, with crocin I accounting for 83%, 93%, and 92% of the total crocins content in S5, S10, and S15, respectively. As shown in Figure 5A, the highest crocin I concentration in leaves reached 5752 ng/g fresh weight (FW). In the fruits, crocin I was the predominant form, representing 97%, 98%, and 99% of total crocins in S5, S10, and S15, respectively. The highest crocin I content in fruits reached 37,132 ng/g FW (Figure 5B). The retention times of trans-crocin I and cis-crocin I were 6.03 and 7.31 min, respectively (Figure 5C). Due to the sterility of the fruiting transgenic plants, LC-MS/MS data for the T1 generation were not available. However, earlier qualitative analyses of leaf samples from lines S7, S13, S5, and S10 showed that S7 and S13 accumulated significantly higher crocin levels than S5 and S10 (Figure 5D). These results suggest that continued screening of positive transgenic lines may yield plants with even higher crocin content.

## 3. Discussion

This study constructed a single vector carrying four genes for crocins biosynthesis. In *S. lycopersicum* fruits, crocin I accounted for 97–99% of total crocins. Compared to wild-type *S. lycopersicum*, the transgenic *S. lycopersicum* exhibit a golden-red hue, although the golden colouration from crocins was still overshadowed by the red of lycopene. Unlike the three fruiting lines, *S. lycopersicum* lines S13 and S7 exhibited noticeable golden colouration in leaves. Nevertheless, this indicates that the potential of this vector for crocin synthesis in *S. lycopersicum* remains underexploited. Subsequent efforts could involve the screening of more positive plants and utilizing trait segregation in the T1 generation to obtain lines with higher content.

Notably, by transforming the two downstream glycosyltransferase genes *GjUGT74F8* and *GjUGT94E13*, this study significantly improved substrate conversion efficiency, addressing the issue of a low proportion of the main active component crocin I in transgenic plants during crocin synthesis [23]. In *N*. *benthamiana*, expressing only the carotenoid cleavage dioxygenase (CCD) can synthesize crocin II and a small amount of crocin I. However, *S. lycopersicum*’s endogenous glycosyltransferases likely function differently from those in *N*. *benthamiana*. When the expression level of *GjUGT74F8* was lower than *GjUGT94E13*, the content of crocin II was too low to be detected in leaves. In *S. lycopersicum* fruit, crocin I constituted the highest proportion of total crocins, reaching up to 99%. If the expression level of *GjUGT74F8* was dominant, the proportion of crocin II in transgenic *S. lycopersicum* would be expected to increase substantially. Transforming *GjUGT74F8* or *GjUGT94E13* alone into *N*. *benthamiana* or *S. lycopersicum* could yield transgenic lines producing high proportions of crocin I or III, respectively, which holds significant value for monomer extraction and purification.

Based on this experimental analysis, the current vector also has room for optimization. For instance, fruit-specific promoters should be used in *S. lycopersicum*, particularly for driving the expression of the key enzyme CCD [26,27]. Furthermore, incorporating key enzyme genes in the carotenoid biosynthesis pathway is crucial, such as adding lycopene β-cyclase (LCYB) to promote the conversion of lycopene to β-carotene, or adding PSY, the rate-limiting enzyme for carotenoid synthesis. Carotenoids and chlorophyll compete for substrates, potentially affecting chlorophyll synthesis, inhibiting plant growth, and reducing downstream conversion efficiency. Crocin synthesis consumes carotenoids, which act as photoprotective factors, and carotenoids compete with chlorophyll for substrates. This substrate competition might explain the occurrence of albino seedlings in *S. lycopersicum* lines. Subsequently, this vector was also transformed into lettuce, resulting in a large number of albino seedlings (unpublished), which might be related to lettuce’s inherently low chlorophyll content. Therefore, future modifications of vectors for crocin synthesis in green tissues could consider introducing the key enzyme GGPPS for both carotenoid and chlorophyll synthesis. Rationally engineered NtGGPPS1 has been shown to simultaneously and significantly increase carotenoid and chlorophyll content in *N*. *benthamiana* [28]. Additionally, the rate-limiting enzymes of the MVA pathway, 3-hydroxy-3-methylglutaryl coenzyme A reductase (HMGR), and the MEP pathway, 1-deoxy-D-xylulose-5-phosphate synthase (DXS), could be incorporated.

Efficient genes play a key role in enabling the host plant to compete for substrates to enhance the synthesis of target products. CCDs, catalyzing the first step of crocin formation, undoubtedly hold a core position in crocin biosynthesis. Besides the carotenoid cleavage dioxygenases from *Crocus sativus* and the GjCCD4a from *G. jasminoides* used in this study, previous research has shown that CCDs from *Bixa orellana*, *Buddleja davidii*, and *Crocus ancyrensis* can cleave carotenoids to produce crocetin dialdehyde [29]. Many CCDs have been verified to cleave lycopene, β-carotene, or zeaxanthin. It was initially believed that CsCCD2L from saffron could only cleave zeaxanthin, but recent studies indicate CsCCD2L also cleaves β-carotene and lycopene, albeit with extremely low affinity. In comparison, GjCCD4a exhibits higher affinity for the upstream β-carotene [30,31]. This might explain why *Nicotiana benthamiana* overexpressing *GjCCD4a* contains higher levels of various crocin components than *Nicotiana benthamiana* overexpressing *CsCCD2L*. Therefore, GjCCD4a is more suitable for crocin synthesis in most plants. Based on this, crocin yield could be increased by boosting β-carotene content in the host plant. Besides introducing foreign genes, direct modification of the host is possible, such as knocking out the α-carotene branch or creating high-β-carotene *S. lycopersicum* germplasm through multi-site mutations, drawing from β-carotene mutants [31,32]. Different CCDs can be selected for different hosts; if a host has higher zeaxanthin content, CsCCD2L could also be used. Future efforts could involve engineering CCDs based on the host; for example, CCDs with higher affinity for lycopene have not yet been reported.

Significant progress has been made in the heterologous synthesis of crocins in plants, but practical extraction and application still face challenges. To replace saffron as a source for extraction, it is essential first to achieve high and stable expression of crocin biosynthetic genes in host plants. This requires a large number of transgenic experiments for the screening of transgenic lines in order to obtain strains with optimal performance and favourable growth characteristics. Second, the growth rate and biomass of the host plant are crucial. Selecting suitable host plants that require less land resources and yield more extractable material is particularly important. The relatively rare golden colour of crocins, combined with their high economic value, makes them highly meaningful for colour breeding. The future exploration of synthetic biology for producing high-value pigment crocins in plant hosts and their application in developing personalized horticultural varieties remains valuable.

*S. lycopersicum*, with high content in this study, died due to diseases and other reasons during planting. Through a large number of genetic transformation experiments and the screening of homozygous offspring, we expect to obtain *S. lycopersicum* plants with higher crocin content. Additionally, the transgenic plants exhibit issues with fruit development. To address the fertility problems in these transgenic lines, they will be crossbred with tomato germplasms that demonstrate normal development and high β-carotene content. The successful expression of crocin in *S. lycopersicum*, a model crop, provides a reference for crocin in healthy colour breeding of fruits and vegetables. In addition to *S. lycopersicum*, there are many plants rich in carotenoid, especially some edible crops, such as carrots, corn, cucumbers, etc., which can not only convert carotenoid from edible parts into saffron but also can be used for extracting saffron from nonedible parts. Citrus, mango, pineapple, etc., can also be considered to be transferred, which can not only enrich the colour but also improve the health function of fruits.

## 4. Materials and Methods

### 4.1. Plant Material, Chemicals and Strains

*Solanum lycopersicum* L. cv. ‘Micro-Tom’ *S. lycopersicumwas* was used for genetic transformation. The *Escherichia coli* strains DH5α andXL10-Gold, as well as *Agrobacterium tumefaciens* strain GV3101, were purchased from WeidiBio (Shanghai, China).

### 4.2. Multigene Expression Vector Construction

We obtained four genes of crocins synthesis through chemical synthesis, including *GjCCD4a*, *GjALDH2C3*, *GjUGT74F8*, and *GjUGT94E13*. In the second round of gene assembly, the ligated dual-gene fragments were individually connected to the CaMV 35S promoter with the HSP terminator and the *Arabidopsis thaliana UBQ10* promoter with the E9 terminator, respectively, and assembled into the pCAMBIA1300 vector, generating two dual-gene expression cassettes: 35S:GjALDH2C2-P2A-GjUGT94E13:Thsp and UBQ10:GjCCD4a-P2A-GjUGT74F8:Te9. In the third round, the two gene expression cassettes, 35S::GjALDH2C3-P2A-GjUGT94E13::Thsp and AtUBQ10::GjCCD4a-P2A-GjUGT74F8::Te9, were assembled into the pCAMBIA1300 vector. The resulting multigene vector, named AU-CU, had the following structure: 35S::GjALDH2C3-P2A-GjUGT94E13::Thsp::AtUBQ10::GjCCD4a-P2A-GjUGT74F8::Te9. All primers used for multigene vector construction are listed in the Table S1.

### 4.3. Transient Expression

An aliquot (200 μL) of *A. tumefaciens* GV3101 harbouring AU-CU was inoculated into 3 mL YEB medium containing the appropriate antibiotics and incubated overnight at 28 °C with shaking (180 rpm). The culture was transferred to 20 mL fresh YEB (supplemented with 50 μg/mL kanamycin, 25 μg/mL rifampicin, 40 μM acetosyringone, and 10 mM MES) and grown for 4–6 h. Cells were collected by centrifugation (4000× *g*, 10 min), washed twice with half-volume infiltration buffer (10 mM MES, 200 μM acetosyringone, 10 mM MgCl_2_), and resuspended in infiltration buffer to OD_600_ = 0.8. After a 2–4 h dark incubation, suspensions were infiltrated into *N*. *benthamiana* leaves and *S. lycopersicum* fruits. Infiltrated tissues were kept in darkness for 24 h and then maintained under a 16 h light/8 h dark photoperiod at 25 °C. Samples were collected 5 days post-infiltration.

### 4.4. S. lycopersicum Plants Transformation

Sterilize Micro-Tom seeds in 10% NaClO for 10 min, followed by three rinses with sterile distilled water. Subsequently, immerse the seeds in 75% (*v/v*) ethanol for 30 s, and then rinse thoroughly four times with sterile distilled water. After sterilization, place the seeds on sterile filter paper to dry. Sow the seeds on solid MS medium and germinate under standard growth conditions. Use leaves, cotyledons, and hypocotyls of Micro-Tom plants as explants. Cut the leaves and cotyledons into pieces, approximately 0.5 cm × 0.5 cm in size, and cut the hypocotyls into segments, approximately 0.5 to 0.6 cm in length. Then, transfer to the pre-culture medium (MS with 1 mg/L 6-BA + 0.2 mg/L NAA) for pre-culturing for 24 h. After pre-culturing, co-culture the explants with strain GV3101 carrying AU-CU vector for 30 min and then transfer to the co-culture medium (MS with 1 mg/L 6-BA + 0.2 mg/L NAA + 0.1 mM AS) for 2 days. Then, transfer the explants to the selection medium (2.0 mg/L ZT, 0.2 mg/L IAA, 10 mg/L Hyg, and 500 mg/L carbenicillin). After sprouting, transfer the shoots to the elongation medium (1.0 mg/L ZT, 0.05 mg/L IAA, 500 mg/L Cef, 10 mg/L Hyg, and 500 mg/L carbenicillin) to grow regenerated shoots to a length of 1 cm. Finally, transfer the shoots to the rooting medium, which consists of 1/2 strength MS medium containing 0.1 mg/L IAA, 10 mg/L Hyg, and 300 mg/L carbenicillin. Rooted plantlets were subsequently acclimatized and transferred to soil under greenhouse conditions.

### 4.5. PCR Detection of Transgenic

Genomic DNA was extracted from leaves of hygromycin-resistant and wild-type plants using a commercial Plant Genomic DNA Kit. Gene-specific primers were used to amplify *GjCCD4a*, *GjALDH2C3*, *GjUGT94E13*, and *GjUGT74F8* to verify genomic integration by PCR. All the primers used for PCR detection are shown in Table S2.

### 4.6. Quantitative Detection of Gene Expression

In order to determine the relative expression levels of the four genes in wild-type and transgenic *S. lycopersicum*. Total RNA was extracted from leaves of wild-type and transgenic plants using a CWBIO RNA extraction kit. The extracted RNA was subjected to reverse transcription using HiScript III 1st Strand cDNA Synthesis Kit (+gDNA wiper) (Nanjing Vazyme Biotech Co., Ltd., Nanjing, China), which enabled the removal of genomic DNA contamination during the process. The obtained first-strand cDNA was then subjected to quantitative real-time PCR (qRT-PCR) using Taq Pro Universal SYBR qPCR Master Mix (Nanjing Vazyme Biotech Co., Ltd., Nanjing, China) on a BIO-RAD CFX96 Real-Time System (Hercules, CA, USA). *Actin* gene was used as a reference gene to normalize the gene expression data. Conditions for qRT-PCR cycling were 95 °C for 30 s followed by 40 cycles of denaturation at 95 °C for 3 s and annealing/extension at 55 °C for 10 s. The primers used for qRT-PCR were listed in Table S3.

### 4.7. Analysis of Mogrosides by HPLC-MS/MS

The entire *S. lycopersicum* fruit was ground, and 0.2 g of the sample was weighed. Then, 20 mL of 80% methanol was added. *S. lycopersicum*. Perform ultrasonic extraction in an ice bath for 1 h and replenish the weight loss. Centrifuge at 6000 rpm for 10 min, collect the supernatant with a syringe, filter it through a membrane, and set aside for later use. HPLC-MS/MS analysis was performed according to the method described by Xie et al. [11].

For quantitative analysis of *S. lycopersicum* fruits and leaves, an Agilent Poroshell 120 SB-C18 column (100 mm × 2.1 mm, 2.7 μm) was employed (Santa Clara, CA, USA). For transient samples and qualitative analysis of *S. lycopersicum* leaves, an Agilent Poroshell 120 SB-C18 column (100 mm × 3.0 mm, 2.7 μm) was used. Both columns maintained at 30 °C. The mass spectrometry system comprised an AB Sciex 4500 QTRAP (Framingham, MA, USA) equipped with an electrospray ionization source (ESI), with ion source temperature at 550 °C and dwell time at 100 ms. In negative ion mode, the entrance potential (EP) and collision cell exit potential (CXP) were −10 V and −15 V, respectively; in positive ion mode, the EP and CXP were both 10 V. Electrospray voltage was set at 5.5 kV and −4.5 kV for positive and negative ion modes, respectively. Nebulizing gases (GS1 and GS2) were pressurized at 55 psi each, with curtain gas (CUR) at 35 psi. Mobile phase A consisted of 0.1% (*v/v*) formic acid in water, and mobile phase B was acetonitrile, delivered at 0.3 mL/min. The HPLC conditions of mogrosides analysis were as follows: 5% B for 0 min; 10% B for 1 min; 40% B for 5 min; 95% B for 2 min; maintain at 95% B for 2 min; 5% B for 10s; and 5% B for 4 min 10 s. The analytical parameters for crocetin and crocins I–V determined by HPLC-MS/MS are summarized in Table 1.

## Figures and Tables

**Table 1:** The HPLC-MS/MS parameters for crocins and crocetin.

Analytes	Molecular Weight	Q1 (*m*/*z*)	Q3 (*m*/*z*)	DP (V)	CE (V)
Crocin I	976.7	999.3	675.0; 347.0	120	50
Crocin II	814.8	837.3	675.0; 347.0	120	50
Crocin III	652.7	675.3	347.0; 351.2	120	45
Crocin IV	652.7	675.3	347.0; 351.2	120	35
Crocin V	490.5	513.3	351.3	120	50
Crocetin	328.4	327.1	239.1; 119.2	−60	−20

## Data Availability

The authors confirm that all data in this experiment are available in the main text and Supplementary data.

## Supplementary Materials

The following supporting information can be downloaded at https://www.mdpi.com/article/10.3390/ijms26209984/s1.
