# Supplementary material for: Heterologous Biosynthesis of Crocin I in Solanum lycopersicum L
Source: Int J Mol Sci. 2025 Oct 14;26(20):9984. doi: 10.3390/ijms26209984 (PMC12564229; doi:10.3390/ijms26209984)
Supplement: Supplementary file 1 [file ijms-26-09984-s001.zip › ijms-3818101-supplementary.pdf]

SUPPLEMENTARY INFORMATION

This file contains the Supplementary Figures and Supplementary Tables corresponding to the manuscript: **"Heterologous Biosynthesis of Crocin I in *Solanum lycopersicum* L."**

Lei Xie [1,†], Jingjing Liao [2,†], Chongnan Wang [1], Xunli Jia [1], Yimei Zang [1], Changming Mo [3,4], Xiaojun Ma [1,*] and Zuliang Luo [1,4,*]

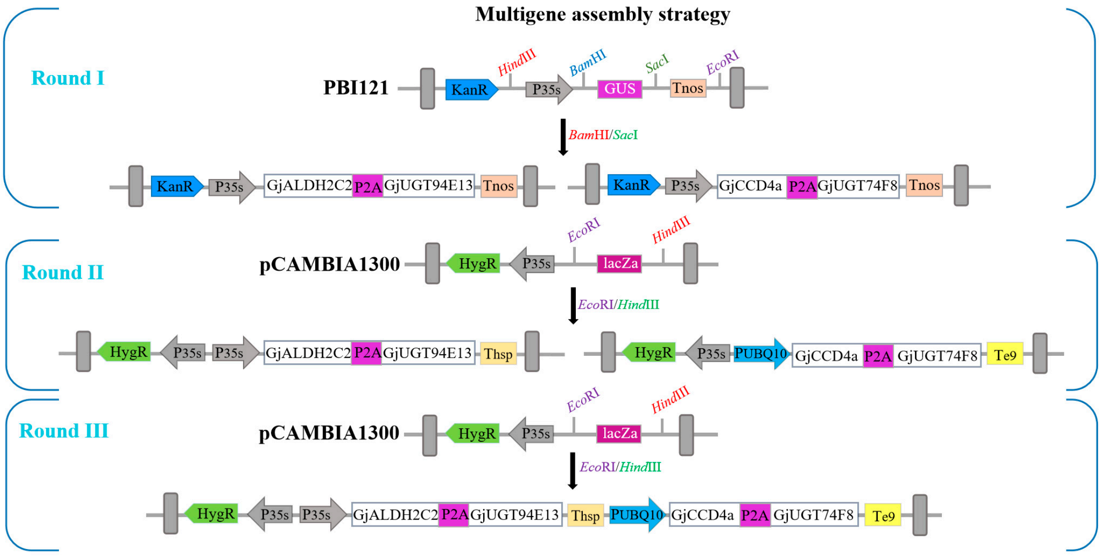

Figure S1. Construction of multigene vector with 4 crocins biosynthesis genes in pCAMBIA1300. a) Flowchart of multigene cassette assembly. The amino acid sequence of P2A is GSGATNFSLLKQAGDVEENPGP. BamHI, SacI, HindIII and EcoRI are restriction enzyme sites. (b) Amplification of specific fragments of the *GjALDH* (1512 bp), *GjUGT94E13* (1365 bp), *GjCCD4a* (1863 bp), *GjUGT74F8* (1392 bp)and Hyg (392 bp) genes from the AU-CU vector. Included is a DNA marker (2000 bp).

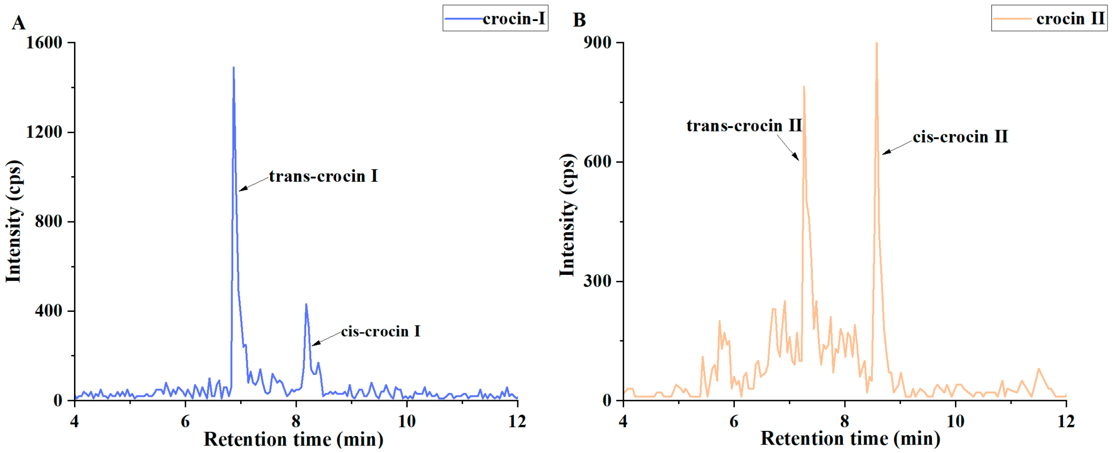

Figure S2. Transient transformation of tomato fruit with AU-CU vector. A. Extracted ion chromatograms of crocin I. B. Extracted ion chromatograms of crocin II.

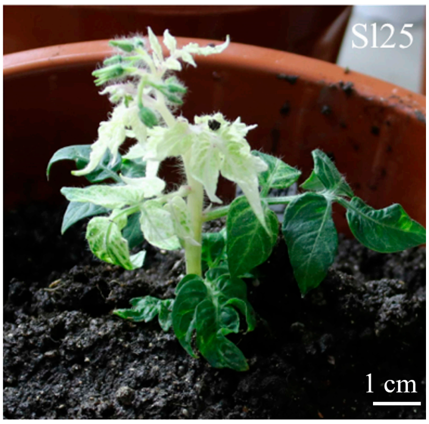

Figure S3. Transgenic albino tomato.

Table S1. Primers used for multigene vector construction

Primers used for double gene fragment ligation

Primer	Sequences (5' to 3')
121-GjALDH2C3-F	acgggggactctagaggatccATGGCCGTCCAAAGCAACG
2AGjUGT94E13-F	caggctggagacgtggaggagaaccctggacctATGAAGGTACTAATGTTGCCATGG
2A(GjUGT94E13)-2F	ggaagcggagctactaacttcagcctgctgaagCAGGCTGGAGACGTGGAGG
121-GjUGT94E13-R	cgatcggggaaattcgagctcTTACTTATCGTCGTCATCCTTGTAATC
GjALDH2C2-2A-R	cttccCAGCCAAGGTGAATTATAAATGGG
GjALDH2C2-2A-F	aattcaccttggctgGGAAGCGGAGCTACTAACTTCAGC
121-GjCCD4a-F	acgggggactctagaggatccATGGATGCTTTCTCTTCCTCTTTC
2AGjUGT74F8-F	gccggtgacgttgaagagaacccaggtccaATGAAGGTACTAATGTTGCCATGG
2A(GjUGT74F8)-2F	ggtgccactaatttttctctcttgaaacagGCCGGTGACGTTGAAGAGAA
121-GjUGT74F8-R	cgatcggggaaattcgagctcCTAATGGTGATGGTGATGATGGTT
GjCCD4a-2A-R	aattagtggcaccTGGACCTGGGTTCTCTTCAACG
GjCCD4a-2A-F	aggtccaGGTGCCACTAATTTTTCTCTCTTGA

The highlighted red portion represents the introduced P2A peptide sequence; underlined sections indicate restriction enzyme cleavage sites; the remaining black lowercase text denotes homologous arm sequences.

Primers used for the dual-gene expression cassettes

Primer	Sequences (5' to 3')
AtUBQ10-F	GTCGACGAGTCAGTAATAAACGGC
AtUBQ10-R	CTGTTAATCAGAAAAACTCAGATTAATCG
1300-35S-F	ctatgacatgattacgaattcTGAGACTTTTCAACAAAGGGTAATATC
GjUGT94E13-Thsp-R	TTACTTATCGTCGTCATCCTTGTAATC
GjUGT94E13-Thsp-F	ggatgacgacgataagtaaATATGAAGATGAAGATGAAATATTTGGTG
1300-Thsp-R	acgacggccagtgccaagcttCTTATCTTTAATCATATTCCATAGTCCATACC
1300-UBQ10-F	ctatgacatgattacgaattcGTCGACGAGTCAGTAATAAACGGC
PUBQ10-GjCCD4a-R	gaagagaaagcatccatCTGTTAATCAGAAAAACTCAGATTAATCG
PUBQ10-GjCCD4a-F	cagATGGATGCTTTCTCTTCCTCTTTC
GjUGT74F8-Te9-R	ctcgaactagtCTAATGGTGATGGTGATGATGGTT
GjUGT74F8-Te9-F	caccattagACTAGTTCGAGCTTTCGTTCGTATC
1300-Te9-R	acgacggccagtgccaagcttTTGATGCATGTTGTCAATCAATTG

The underlined sections indicate restriction enzyme cleavage sites, while the remaining black lowercase portions represent homologous arm sequences.

Primers for final gene expression vector construction	
**Primers**	**Sequences (5' to 3')**
1300-35S-F	ctatgacatgattacgaattcTGAGACTTTTCAACAAAGGGTAATATC
Thsp-PUBQ10-R	ctgactcgtcgacCTTATCTTTAATCATATTCCATAGTCCATACC
Thsp-PUBQ10-F	agataagGTCGACGAGTCAGTAATAAACGGC
1300-Te9-R	acgacggccagtgccaagcttTTGATGCATGTTGTCAATCAATTG

The underlined sections indicate restriction enzyme cleavage sites, while the remaining black lowercase portions represent homologous arm sequences.

Table S2. Primers for PCR detection.

Primers	Sequences (5' to 3')
***GjCCD4a*-F**	ATGACTGATTTATTGTGTTTCCTATACG
***GjCCD4a*-R**	TTACAATTTATTGAGCTCACTTTCTCTG
***GjALDH2C3*-F**	ATGGCCGTCCAAAGCAACG
***GjALDH2C3*-R**	TTACAGCCAAGGTGAATTATAAATGG
***GjUGT74F8*-F**	ATGAATTCCAGCAAAGTTCATGTT
***GjUGT74F8*-R**	CTAGTTCTGGGCCTTCTGGCC
***GjUGT94E13*-F**	ATGAAGGTACTAATGTTGCCATGG
***GjUGT94E13*-R**	TTAACAGCCCCCGTTGTTCTT

Table S3. Primers for qRT-PCR.

Primers	Sequences (5' to 3')
***GjCCD4a*-qF**	CAAGATTGATCTCAAGACTGG
***GjCCD4a*-qR**	GTCTACAACTGCTGCATATAC
***GjALDH2C3*-qF**	GGATTCTCATGTTAAGGTTCC
***GjALDH2C3*-qR**	GGATCTATTGTCTCAAACGTC
***GjUGT94E13*-qF**	GATATTAGAAAAGTCGGGAGC
***GjUGT74F8*-qF**	CAAAGGACTCGGTAGTTTATG
***Leactin-q*F**	CCAGGTATTGCTGATAGAATGAG
***Leactin-q*R**	GAGCCTCCAATCCAGACAC
